# Quercetin attenuates lipopolysaccharide-induced hepatic inflammation by modulating autophagy and necroptosis

**DOI:** 10.1016/j.psj.2024.103719

**Published:** 2024-04-02

**Authors:** Jinhai Yu, Rong Fu, Amin Buhe, Bing Xu

**Affiliations:** ⁎ Camellia Research Institute, The Innovation Institute of Agricultural Technology, Department of Life Science, Shangrao Normal University, Shangrao 334001, China; † Department of Literature and Media, Shangrao Normal University, Shangrao 334001, China; ‡ Department of Cancer Surgery, Beijing Shijitan Hospital Affiliated with Capital Medical University, Beijing 100038, China

**Keywords:** quercetin, endotoxin, liver, injury, programmed cell death

## Abstract

Lipopolysaccharide (**LPS**) from Gram-negative bacteria initially induces liver inflammation with proinflammatory cytokines expressions. However, the underlying hepatoprotective mechanism of quercetin on LPS-induced hepatic inflammation remains unclear. Specific pathogen-free chicken embryos (*n* = 120) were allocated control vehicle, PBS with or without ethanol vehicle, LPS (125 ng/egg) with or without quercetin treatment (10, 20, or 40 nmol/egg, respectively), quercetin groups (10, 20, or 40 nmol/egg). Fifteen-day-old embryonated eggs were inoculated abovementioned solutions via the allantoic cavity. At embryonic d 19, the livers of the embryos were collected for histopathological examination, RNA extraction, real-time polymerase chain reaction, and immunohistochemistry investigation. We found that the liver presented inflammatory response (heterophils infiltration) after LPS induction. The LPS-induced mRNA expressions of inflammation-related factors (TLR4, TNFα, IL-1β, IL-10, IL-6, MYD88, NF-κB1, p38, and MMP3) were upregulated after LPS induction when compared with the PBS group, while quercetin could downregulate these expressions as compared with the LPS group. Quercetin significantly decreased the immunopositivity to TLR4 and MMP3 in the treatment group when compared with the LPS group. Quercetin could significantly downregulate the mRNA expressions of autophagy-related genes (ATG5, ATG7, Beclin-1, LC3A, and LC3B) and necroptosis-related genes (Fas, Bcl-2, Drp1, and RIPK1) after LPS induction. Quercetin significantly decreased the immunopositivity to LC3 in the treatment group when compared with the LPS group; meanwhile, quercetin significantly decreased the protein expressions of LC3-I, LC3-II, and the rate of LC3-II/LC3-I. In conclusions, quercetin can alleviate hepatic inflammation induced by LPS through modulating autophagy and necroptosis.

## INTRODUCTION

*Salmonella enterica subspecies enterica* serovar Typhimurium is a leading foodborne illness worldwide (Hendriksen et al., 2011), which had important role in public health and meat products (Zeng et al., 2019; Yang et al., 2020). However, cumulative evidence showed that *Salmonella enterica* Typhimurium presented the multidrug resistance (Yu, et al., 2008; Woh et al., 2021; Zhang, et al., 2021). It is imperative to search alternative medicine to deal with this situation. Lipopolysaccharide (**LPS**), endotoxin from Gram-negative bacteria (including *Salmonella spp.*), induces acute multiple organ injures or inflammation including liver and heart (Shaker and Sahib, 2023). The liver is the largest and a highly dynamic metabolic organ that plays critical roles in nutrition and drug/xenobiotic metabolism, and detoxification. Previous studies indicated that the mRNA expressions of tumor necrosis factor alpha (**TNFα**), interleukin-1β (**IL-1β**), IL-6, toll-like receptor 4 (**TLR4**), myeloid differentiation primary response gene 88 (**MYD88**), and nuclear factor kappa B (**NF-κB**) increased after LPS induction in the livers of piglets (Zhou et al., 2022b; Duan et al., 2023) and mice (Zhou et al., 2022a). Another study demonstrated that the protein expressions of TLR4, TNFα, and IL-6 increased after LPS stimulation in the liver of weaning piglets (Soldatenko et al., 2022). In addition, the mRNA expressions and protein levels of IL-1β, IL-18, and NOD-like receptor family pyrin domain-containing 3 increased after LPS stimulation in the liver of mice (Dou et al., 2022). Many phytochemicals had been used to alleviate LPS-induced acute liver injury, such as artemisitene (Fan et al., 2023), salidroside (Meng et al., 2024), resveratrol (Wu et al., 2023), Astragaloside IV (Sun et al., 2023), and dihydrosanguinarine (Xiang et al., 2022); Quercetin, a natural flavonoid, has been used as an alternative supplement for its anti-inflammatory, anti-oxidative properties, and anti-cancer activities (Maugeri et al., 2023). One study had found that quercetin attenuated enterotoxigenic *Escherichia coli*-caused inflammation and necroptosis in the intestinal porcine epithelial cell line (Xiao et al., 2023). Nevertheless, the protective effect of quercetin alleviates *Salmonella enterica* Typhimurium LPS caused liver inflammation and programmed cell death in chicken embryos remain unclear.

Autophagy is a highly conserved process in eukaryotic cell. Autophagy regulated catabolic process for degrading and recycling damaged or unnecessary organelles, which plays crucial roles in cell survival. The interaction of hepatic autophagy and programmed cell death (pyroptosis, apoptosis, and necroptosis) plays an important role in the physiology and pathology (Zhao et al., 2022). Previous study showed that the autophagy-related protein levels (high-mobility group box 1 protein) increased in the liver of rats after LPS induction (Hagiwara, et al., 2012), another study indicated that LPS induced the protein levels of microtubule associated protein 1 light chain 3 beta II(**LC3II**) increased in the murine livers and hepatocytes (Chung, et al., 2017). One study revealed that the protein expressions of LC3II and LC3I increased in the liver of chick after *salmonella* enteritidis infection (Shen, et al., 2019). Previous study indicated that taraxasterol alleviates aflatoxin B-induced liver damage in broiler chickens via regulation of autophagy (Sang, et al., 2023). However, there is no report of underlying protective mechanism on quercetin alleviate the hepatic autophagy after LPS induction in chicken embryos.

Necroptosis, an inflammatory programmed cell death, is not only a caspase independent form, but also is a lytic form in which the cellular contents are released in the inflammatory response, and these contents serve as damage associated molecular patterns (**DAMP**) which are endogenous ligands for pattern recognition receptors. The signal molecule Drp1 and receptor interacting serine/threonine kinase 1 (**RIPK1**) can regulate the necroptosis. TNFα and its receptor are capable of inducing necroptosis directly. Previous study indicated that the mRNA expression and protein levels of RIPK1, RIPK3, mixed lineage kinase domain-like (**MLKL**) (necroptosis indicator) increased after LPS challenge in the liver of piglets (Wang et al., 2023) and chicken (Zhirong, et al., 2021). One study showed that quercetin alleviated the mRNA expression and protein levels of RIPK1, RIPK3, and MLKL induced by Cadmium in the chicken brain (Liu, et al., 2021). Nevertheless, the protective mechanism of quercetin ameliorate hepatic necroptosis after LPS induction remains unclear.

In the present study, LPS was used to establish the hepatic injury and inflammation model of chicken embryos, the balance effects of quercetin on inflammatory response, autophagy, and necroptosis were studied.

## MATERIAL AND METHODS

### Reagents, Chicken Embryos and Experimental Design

LPS from *Salmonella enterica* serotype Typhimurium (*S.* Typhimurium, product number: L7261, Sigma-Aldrich Trading Co. Ltd., Shanghai, China) was dissolved in a phosphate-buffered solution (**PBS**) at 0.625 μg/mL (125 ng/egg). Quercetin (Product number: Q4591, Sigma-Aldrich Trading Co. Ltd., Shanghai, China) was dissolved in 100% ethanol at 50, 100, or 200 μmol/L (10, 20 or 40 nmol/egg). There is ethanol dehydrogenase in the livers of the chickens (Estonius et al., 1990), and the ethanol metabolism increased after 20 min of intra-allantoic injection and then went back to control level after that (Ten Busch, et al., 1997). Hence, quercetin were dissolved by ethanol to increase the solubility.

Because the chick genome demonstrates remarkable evolutionary conservation with mammals, the expression patterns of several genes and proteins are well-conserved between chick and mouse embryos. In addition, injection into the allantoic cavity of chicken embryos was an ideal method to avoid the interaction of environmental LPS and intestinal LPS from gut microorganisms. Therefore, the chicken embryos were selected for the present study. Specific pathogen-free embryos (weight 56.76 ± 3.32 g) were provided by a chicken breeder (Ji'nan SAIS Poultry Co. Ltd., Ji'nan, Shandong, China). The fertilized eggs were individually weighed and divided into 10 groups, each group consisting of 4 replicates with 3 eggs per replicate. The embryos were incubated under standard conditions (temperature: 38°C, humidity: 60–70%). All eggs were candled and weighed at embryonic d 7 and embryonic d 14 to eliminate undeveloped eggs. They were untreated or injected with 0.2 mL/egg of PBS, LPS (125 ng/egg; 0.2 mL/egg), PBS and ethanol (0.2 mL each per egg), quercetin and LPS groups (10, 20 or 40 nmol/egg; 0.2 mL/egg), and quercetin groups (10, 20, or 40 nmol/egg). Each treatment was administered to 15-day old embryonated eggs by injection into the allantoic cavity according to the procedure described by Manders et al. (2021). The weighing, examination, and injection of the treatment solution to the chicken embryos were the same as our previous study (Yu et al., 2022).

At embryonic d 19, the livers of the embryos were collected for histological examination, and RNA extraction for real-time quantitative polymerase chain reaction (**qPCR**). The hepatic tissues for histological examination were processed by the routine method (see below). The sample for PCR was stored in liquid nitrogen until RNA extraction. The study was approved by the University Animal Ethics Committee (SS-202203001).

### Histology

Liver tissues were fixed in 4% paraformaldehyde; dehydrated; embedded in paraffin blocks; sectioned to 3 μm thick sections (model:RM2016, Shanghai Leica Instrumental Ltd., Shanghai, China); mounted on slides; and stained with hematoxylin and eosin following established histology procedures. The slides were scanned by Panoramic DESK (3D HISTECH Ltd., Hungary) with the panoramic scanner software. Case viewer software (3D HISTECH Ltd., Hungary) was used to take pictures.

### Real Time qPCR

The protocols of qPCR were the same as our previous study (Yu et al., 2022). A total of eighteen genes were selected to show the hepatic inflammatory factors, autophagy, and necroptosis (Table 1). Glyceraldehyde -3- phosphate dehydrogenase (GAPDH) was used as the housekeeping gene. The sequence of genes was obtained from the USA National Center for Biotechnology Information web (**NCBI**, https://www.ncbi.nlm.nih.gov/nuccore), and the forward and reverse primers were obtained by Primer-BLAST (https://www.ncbi.nlm.nih.gov/tools/primer-blast).The relative levels of target mRNA expression were calculated using the 2^−∆∆Ct^ method.

**Table 1:** Primers used in real-time quantitative polymerase chain reaction.

Genes name	Primer sequence(5′-3′)		Gene bank ID
**ATG5**	F:CCCATCCCTGGTCCGTAAC;	R:CGGCGGCGTATACGAAGTA	NM_001006409
**ATG7**	F:TGCAGTTTGCTCCCTTCAGT	R:TGGGAAACCTGATGGATCGC	NM_001396468
**Bcl-2**	F: TGGCTGCTTTACTCTTGGGG;	R:TATCTCGCGGTTGTCGTAGC	NM_205339
**Beclin-1**	F:CCGCTGAAGCTCGATACCTC	R:TTCTGGCTGGTGGGATGAAC	NM_001006332.1
**Fas**	F:GTCAGTGCTGCACGAAATGT;	R:AACCTCCAAACCGAGTGCTT	NM_001199487
**Drp1**	F: GGCAGTCACAGCAGCTAACA;	R:GCATCCATGAGATCCAGCTT	NM_001079722
**GAPDH**	F: GAGAAACCAGCCAAGTATGATG;	R: CACAGGAGACAACCTGGTCC	NM_204305
**IL-1β**	F:GCTCAACATTGCGCTGTACC;	R:AGGCGGTAGAAGATGAAGCG	FJ537850
**IL-6**	F:ACGAGGAGAAATGCCTGACG;	R:CTTCAGATTGGCGAGGAGGG	NM_204628
**IL-10**	F:TGCGAGAAGAGGAGCAAAGC	R:AACTCCCCCATGGCTTTGTAG	AJ621254
**LC3B**	F:CTTCTTCCTCCTGGTGAACG;	R:GCACTCCGAAAGTCTCCTGA	NM_001031461
**LC3A**	F:GCATCCAAACAAAATCCCAGTC;	R:AAGCCATCCTCATCCTTCTCCT	XM_040688401
**MMP3**	F:ATCAGGCTCTACAGTGGTG;	R:ATGGGATACATCAAGGCAC	XM_025152201
**NF-κB1**	F:TCAACGCAGGACCTAAAGACAT;	R:GCAGATAGCCAAGTTCAGGATG	NM_001396396
**p38**	F:GGTCGGTGAGCTGGTAAAGG;	R:CGCTTTCAGCTTCTGTCGGA	XM_015296032
**RIPK1**	F:GATCCATTTGCGAAGCTGCC	R:CTTAGGCTAATGGCGCTGGT	NM_204402
**TLR4**	F:GGCTCAACCTCACGTTGGTA;	R:AGTCCGTTCTGAAATGCCGT	KP410249
**TNFα**	F:CCCATCCCTGGTCCGTAAC;	R:CGGCGGCGTATACGAAGTA	MF000729

Abbreviation: ATG5: Autophagy related gene 5; ATG7: Autophagy related gene 7; Bcl-2: B cell CLL/lymphoma 2; Drp1: dynamin 1 like; GAPDH: Glyceraldehyde-3-phosphate dehydrogenase; Fas: Fas cell surface death receptor; IL-1β: Interleukin-1β; IL-6: Interleukin-6; IL-10: Interleukin-10; LC3A: Microtubule associated protein 1 light chain 3 alpha; LC3B: Microtubule associated protein 1 light chain 3 beta; MMP3: Matrix metallopeptidase 3; NF-κB1: Nuclear factor kappa B subunit 1; RIPK1: receptor interacting serine/threonine kinase 1; TLR4: Toll like receptor 4; TNFα: Tumor necrosis factor alpha.

### Immunohistochemistry Investigation

Immunohistochemical investigations were carried out using indirect method of peroxidase with a primary antibody specific for LC3 (anti-LC3, GB11124, Servicebio, Wuhan servicebio technology CO., LTD), TLR4 (anti-TLR4, GB11519, Servicebio), and matrix metallopeptidase 3 (MMP3, anti-MMP3, GB11131, Servicebio). The protocols of immunohistochemiscal investigation were the same as our previous study (Yu et al., 2023).

### Western Blot Analysis

The protocols of Western blotting analysis were the same as our previous study (Yu et al., 2022). The specific antibodies were used to detect LC3-I/LC3-II (1:3000, Servicebio), GAPDH (1:2,000, Servicebio), and the secondary IgG HRP-conjugated antibody (1:5,000; Servicebio). The expression levels of the target proteins were normalized to GAPDH.

### Statistical Analysis

All data were statistically analyzed using the paired T test and ANOVA method by the *SPSS* software (version 16.0, SPSS Inc., Chicago, IL), and were presented as mean ± standard deviation. Differences were considered significant for values of *P* < 0.05.

## RESULTS

### The Protective Effects of Quercetin on Liver After LPS Induction in Chicken Embryos

**Figure 1:**
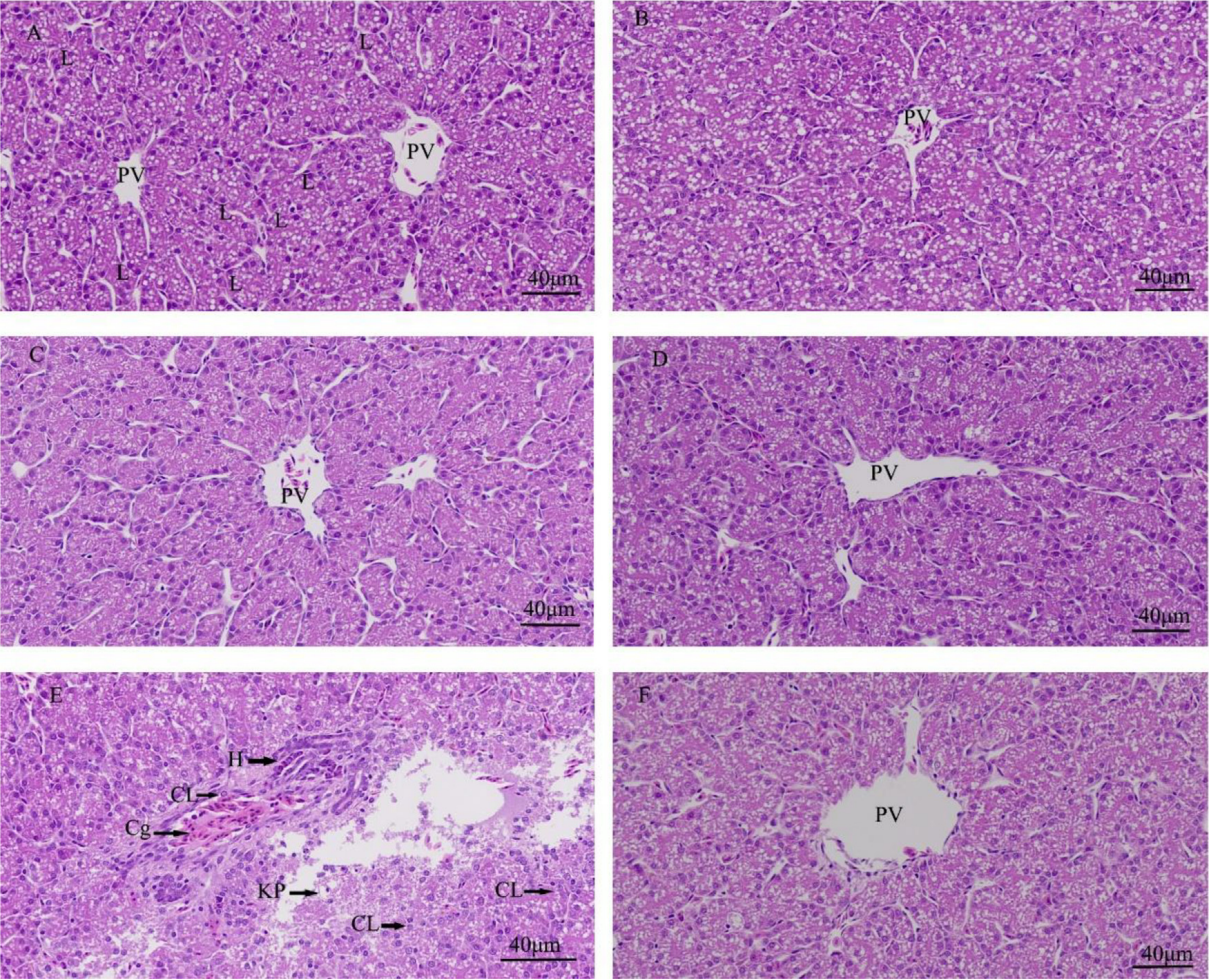
The histopathologic changes of livers induced by LPS and quercetin in chicken embryos. Hematoxylin and eosin stain (400 ×). (A) Control group; (B) PBS group; (C) PBS + ethanol group; (D) 40 nmol Q, 40 nmol quercetin; (E) LPS group (125 ng LPS/egg); (F) 125 ng LPS + 40 nmol Q, LPS (125ng/egg) and quercetin (Q) at 40 nmol/egg. PV:Portal veins; L: Lipid droplets; H: Heterophils; Cg: Congestion; CL: Caryolysis; KP: Karyopycnosis (arrow). Scale bar: 40 μm.

Based on the histopathological analyses, effects of quercetin on LPS-induced hepatic inflammation were studied in the chicken embryos. There was no significant change and no inflammatory response in the control group, the PBS group, the PBS + ethanol group, and the quercetin group (Figures 1A–1D). They all showed fatty liver morphology and there were a variety of lipid droplets in the liver tissues. The hepatic cord was arranged radially, and the nucleus was located in the center of the cell. Hepatocytes was round and full. Nevertheless, there were inflammatory cells infiltration (the heterophils accumulated) and mild congestion near or in portal veins of chicken embryos after LPS induction ((125ng LPS/egg) (Figure 1E). The microstructure of hepatocytes showed that the caryolysis and karyopycnosis occurred, the structure of liver tissue presented extracellular matrix lysis and destruction. There were morphological changes on cytoplasmic lipid droplets or vacuoles after LPS challenge, lipid droplets became smaller, fused and had vague outline. No inflammatory cell infiltration and structure destruction were presented in the LPS + Q group (125 ng LPS/egg + 40 nmol Q/egg) (Figure 1F).

### Quercetin Ameliorates Hepatic Inflammation in Chicken Embryos

**Figure 2:**
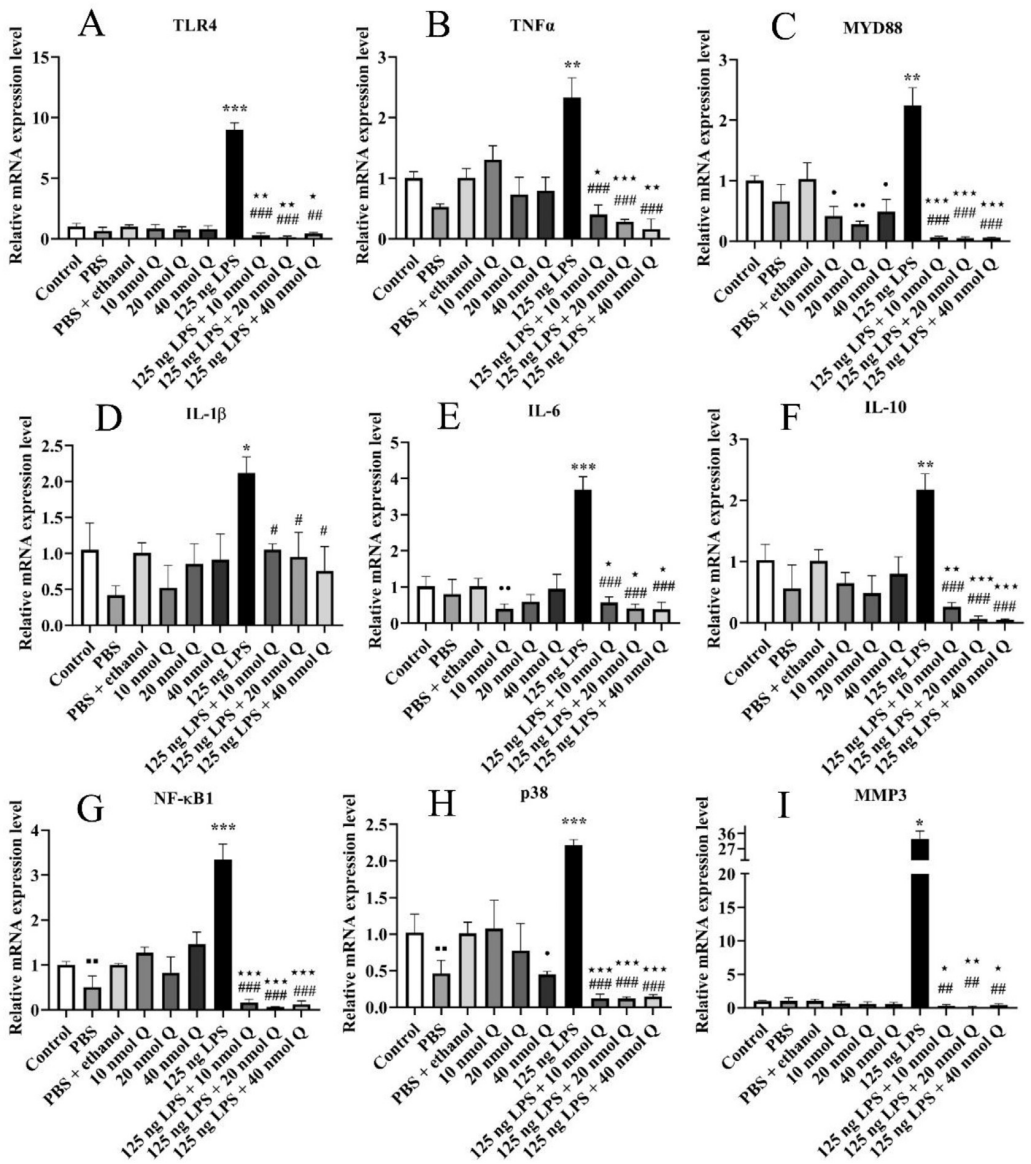
Quercetin downregulated the hepatic mRNA expressions of inflammation-related genes after LPS induction in the chicken embryos. Data are presented as the mean ± SD. * indicates the significant differences between the PBS group and the LPS group; * < 0.05, ** < 0.01, *** < 0.001; # indicates the significant differences between the LPS group and the LPS + Q group; # < 0.05, ## < 0.01, *** < 0.001; ★ indicates the significant differences between the PBS + ethanol group and the LPS + Q group; ★ < 0.05, ★★ < 0.01, ★★★ < 0.001;● indicates the significant differences between the PBS + ethanol group and the Q (quercetin) group; ● < 0.05, ●● < 0.01, ●●● < 0.001.

There was no different mRNA expression in inflammatory-related factors [TLR4, TNFα, MYD88, IL-1β, IL-10, IL-6 (Figures 2A–2F), and MMP3 (Figure 2I)] when compared the PBS group or the PBS + ethanol group with the control group, respectively. The mRNA expressions of NF-κB1 and p38 in the PBS group decreased when compared with the control group (*P* < 0.01) (Figures 2G–2H). Quercetin with 10 nmol significantly downregulated the mRNA expressions of IL-6 when compared with the PBS + ethanol group (*P* < 0.01) (Figure 2E); meanwhile, quercetin with 40 nmol significantly downregulated the mRNA expressions of p38 when compared with that of the PBS + ethanol group (*P* < 0.05) (Figure 2H). The mRNA expressions of MYD88 in the quercetin groups significantly downregulated when compared with the PBS + ethanol group (*P* < 0.05 or *P* < 0.01) (Figure 2C). Quercetin treatment (LPS + Q) group significantly downregulated the mRNA expressions of TLR4, TNFα, MYD88 (Figure 2A–2C), IL-6, IL-10, NF-κB1, p38, and MMP3 (Figure 2E–2I) when compared with that of the PBS + ethanol groups (*P* < 0.05, *P* < 0.01, or *P* < 0.001).

The mRNA expressions of pattern recognition receptor TLR4 (Figure 2A), inflammatory cytokines including MYD88, IL-1β, IL-6, IL-10, TNFα (Figures 2B–2F), nuclear factor NF-κB1 (Figure 2G), mitogen activated protein kinases p38 (Figure 2H), and matrix metallopeptidase MMP3 (Figure 2I) increased by 9.0 folds, 2.2 folds, 2.1 folds, 3.7 folds, 2.2 folds, 2.3 folds, 3.3 folds, 2.2 folds, and 32.7 folds after LPS induction when compared with that of the PBS group, respectively (*P* < 0.05, *P* < 0.01, or *P* < 0.001). Quercetin significantly inhibited the LPS-induced expression of these inflammation-related factors at all doses (*P* < 0.05, *P* < 0.01, or *P* < 0.001).

**Figure 3:**
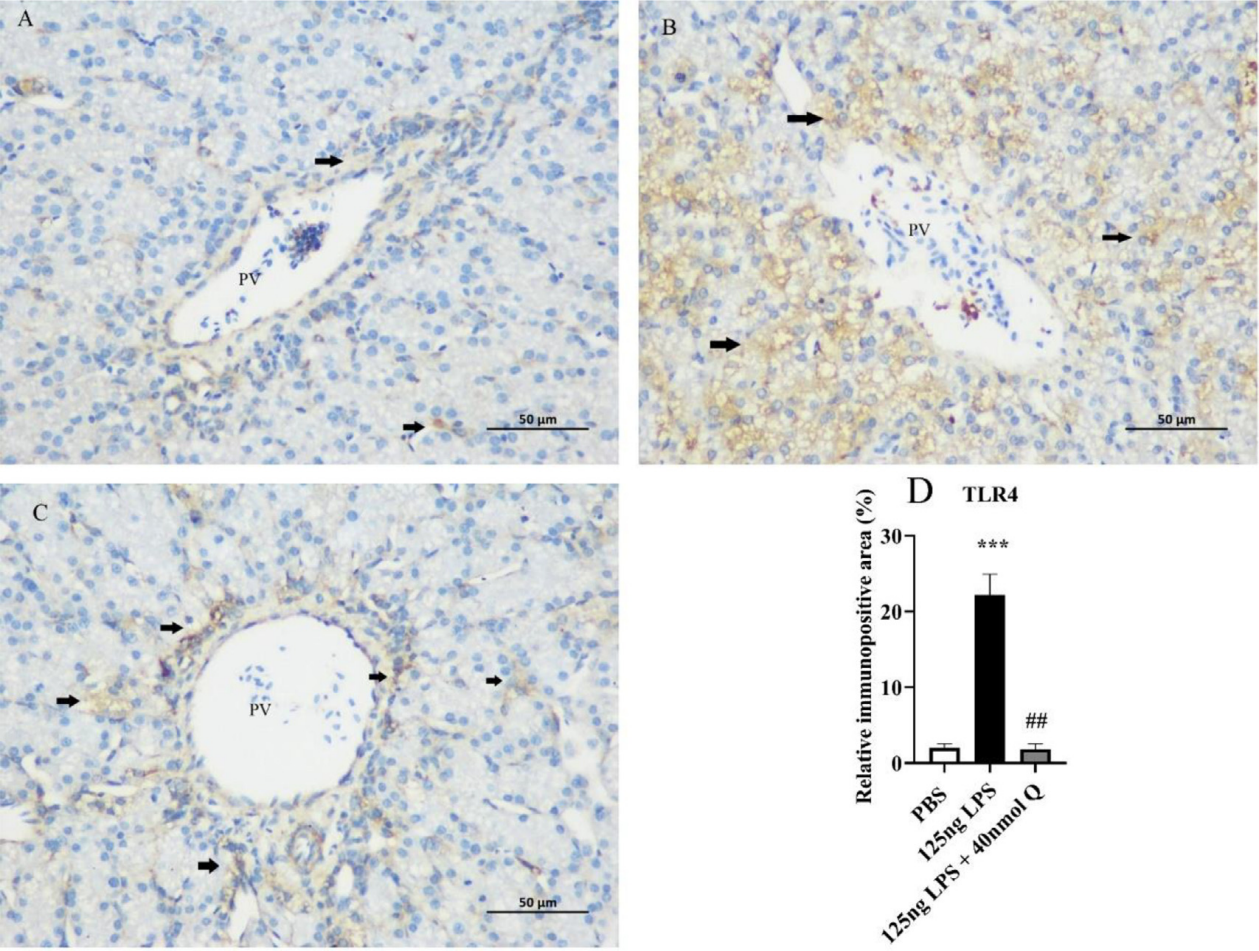
Quercetin alleviates the protein expression of TLR4 induced by LPS in the livers of chicken embryos by immunohistochemical investigation. (A) PBS group; (B) LPS group (125 ng /egg); (C) treatment group (125 ng LPS/egg + 40 nmol quercetin /egg); (D) relative immunopositive area of TLR4. Immunopositivity to TLR4 (arrow, brown to yellow). Scale bar: 50 μm. Data were expressed as the mean ± SD. * indicates the significant differences between the PBS group and the LPS group; ***: *P* < 0.001. # indicates the significant differences between the LPS group and the LPS + Q group; ##: *P* < 0.01.

**Figure 4:**
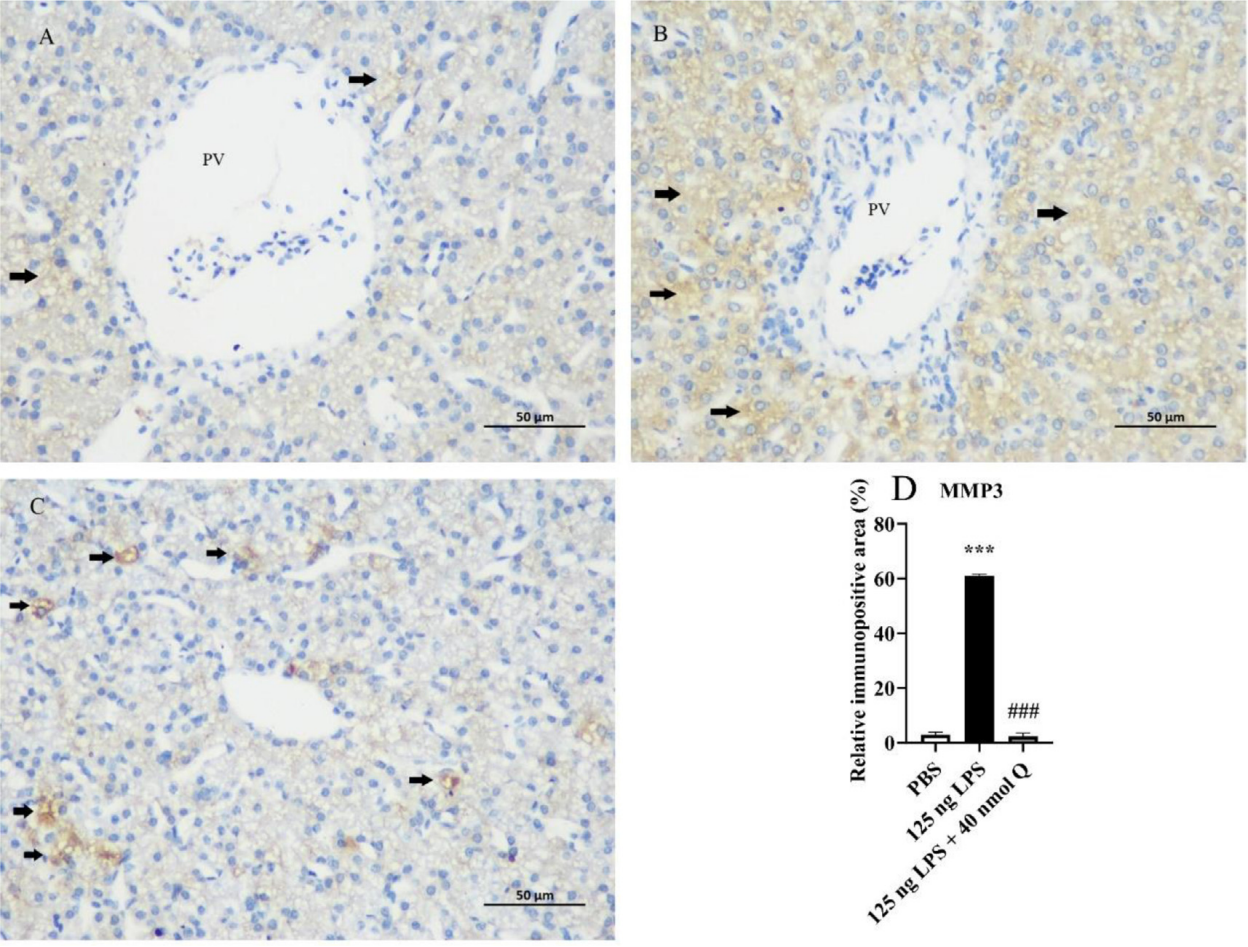
Quercetin alleviates the protein expression of MMP3 induced by LPS in the livers of chicken embryos by immunohistochemical investigation. (A) PBS group; (B) LPS group (125 ng /egg); (C) treatment group (125 ng LPS/egg + 40 nmol quercetin /egg); (D) relative immunopositive area of MMP3. Immunopositivity to MMP3 (arrow, brown to yellow). Scale bar: 50 μm. Data were expressed as the mean ± SD. * indicates the significant differences between the PBS group and the LPS group; ***: *P* < 0.001. # indicates the significant differences between the LPS group and the LPS + Q group; ###: *P* < 0.001.

The immunopositivity of TLR4 and MMP3 in the cytoplasms of hepatocytes significantly increased after LPS induction when compared with that of the PBS group (*P* < 0.01 or *P* < 0.001), whereas the immunopositivity to TLR4 and MMP3 in the LPS + Q group decreased when compared with that of the LPS group (*P* < 0.05 or *P* < 0.001) (Figure 3, Figure 4). These findings revealed that quercetin attenuated LPS-induced hepatic inflammation.

### Quercetin Alleviates Hepatic Inflammation Via Modulating Autophagy and Necroptosis in Chicken Embryos

**Figure 5:**
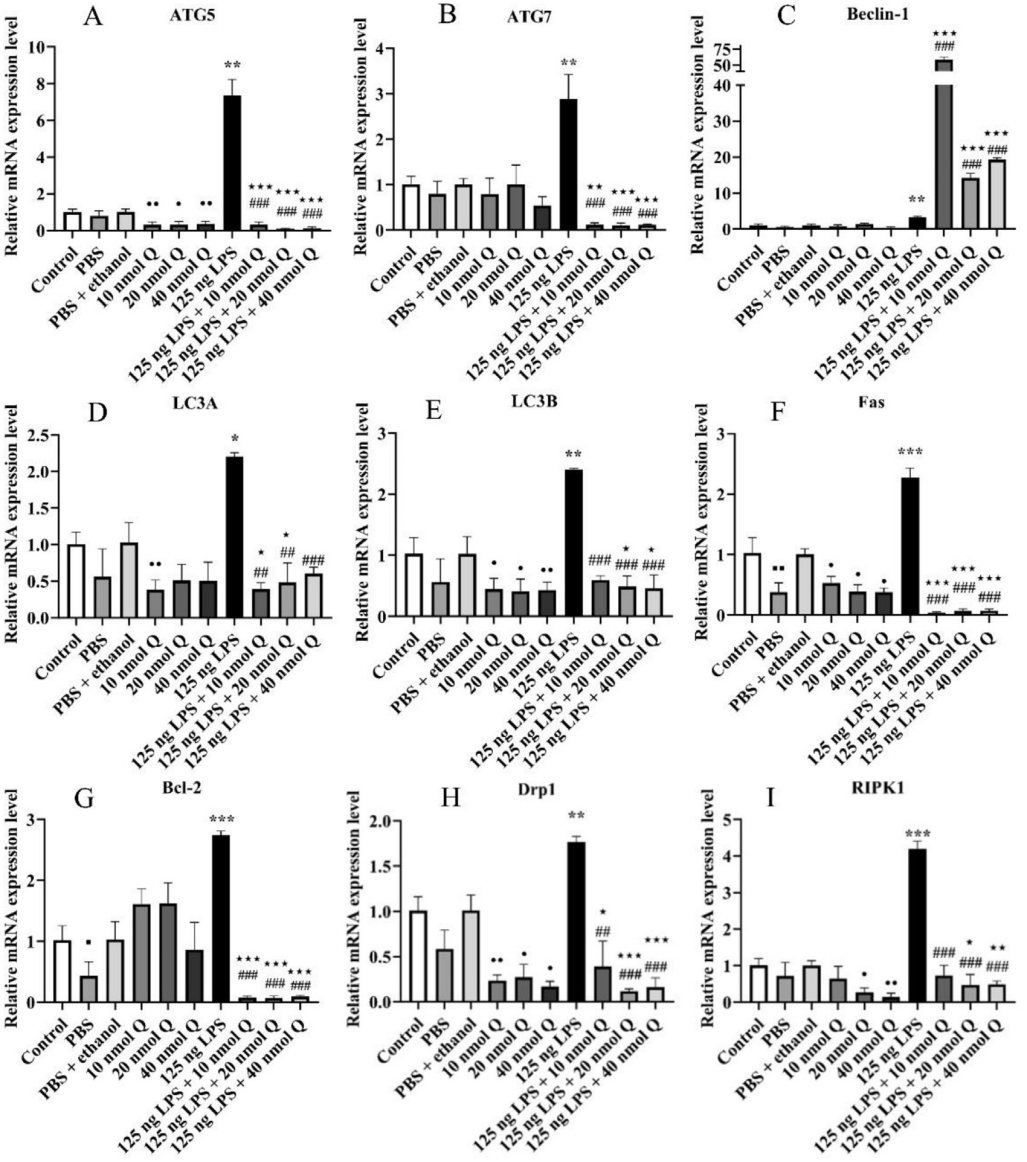
Quercetin attenuates LPS-induced hepatic inflammation by modulating the mRNA expressions of autophagy and programmed cell death-related genes in the chicken embryos. Data are presented as the mean ± SD. * indicates the significant differences between the PBS group and the LPS group; * < 0.05, ** < 0.01, *** < 0.001; # indicates the significant differences between the LPS group and the LPS + Q group; # < 0.05, ## < 0.01, *** < 0.001; ★ indicates the significant differences between the PBS + ethanol group and the LPS + Q group; ★ < 0.05, ★★ < 0.01, ★★★ < 0.001;● indicates the significant differences between the PBS + ethanol group and the Q (quercetin) group; ● < 0.05, ●● < 0.01, ●●● < 0.001.

There was no different mRNA expression in autophagy-related factors (ATG5, ATG7, Beclin-1, LC3A, and LC3B) (Figures 5A–5E) when compared the PBS group or the PBS + ethanol group with the control group, respectively. Quercetin with 10 nmol, 20 nmol or 40 nmol significantly downregulated the mRNA expressions of ATG5 and LC3B when compared with the PBS + ethanol group (*P* < 0.05 or *P* < 0.01) ((Figures 5A and 5E); meanwhile, quercetin with 10 nmol significantly downregulated the mRNA expressions of LC3A when compared with that of the PBS + ethanol group (*P* < 0.01) (Figure 5D). Quercetin treatment (LPS + Q) significantly downregulated the mRNA expressions of ATG5 and ATG7 when compared with that of the PBS + ethanol groups (*P* < 0.01 or *P* < 0.001). Quercetin treatment (125 ng LPS +10 nmol Q or 125 ng LPS +20 nmol Q) significantly downregulated the mRNA expressions of LC3A when compared with that of the PBS + ethanol groups (*P* < 0.05) (Figure 5D). Quercetin treatment (125 ng LPS + 20 nmol Q or 125 ng LPS + 40 nmol Q) significantly downregulated the mRNA expressions of LC3B when compared with that of the PBS + ethanol groups (*P* < 0.05) (Figure 5E).

Autophagy signaling pathway was activated by LPS induction, mRNA expression of ATG5 (Figure 5A), ATG7 (Figure 5B), LC3A (Figure 5D), and LC3B (Figure 5E) markedly increased 7.4, 2.9, 2.2, and 2.4 folds (*P* < 0.05 or *P* < 0.01), respectively. However, quercetin with LPS downregulated the mRNA expressions of ATG5, ATG7, LC3A, and LC3B at 3 doses when compared with the LPS groups (*P* < 0.01 or *P* < 0.001). The mRNA expressions of Beclin-1 were upregulated after LPS induction (*P* < 0.01); meanwhile, quercetin with LPS upregulated the mRNA expressions of Beclin-1 when compare with that of the LPS groups or the PBS + ethanol groups (*P* < 0.001).

Hepatic death ligands Fas, Bcl-2, mitochondria fission factor (**Drp1**), and necroptosis-associated gene (RIPK1) (Figure 5F–5I) were significantly upregulated by LPS, and increased 2.3, 2.7, 1.8, and 4.2-folds (*P* < 0.01 or *P* < 0.001), respectively. Nevertheless, quercetin treatment significantly decreased the mRNA expressions of Fas, Bcl-2, Drp1, and RIPK1, respectively (*P* < 0.01 or *P* < 0.001) (Figures 5F–5I).

**Figure 6:**
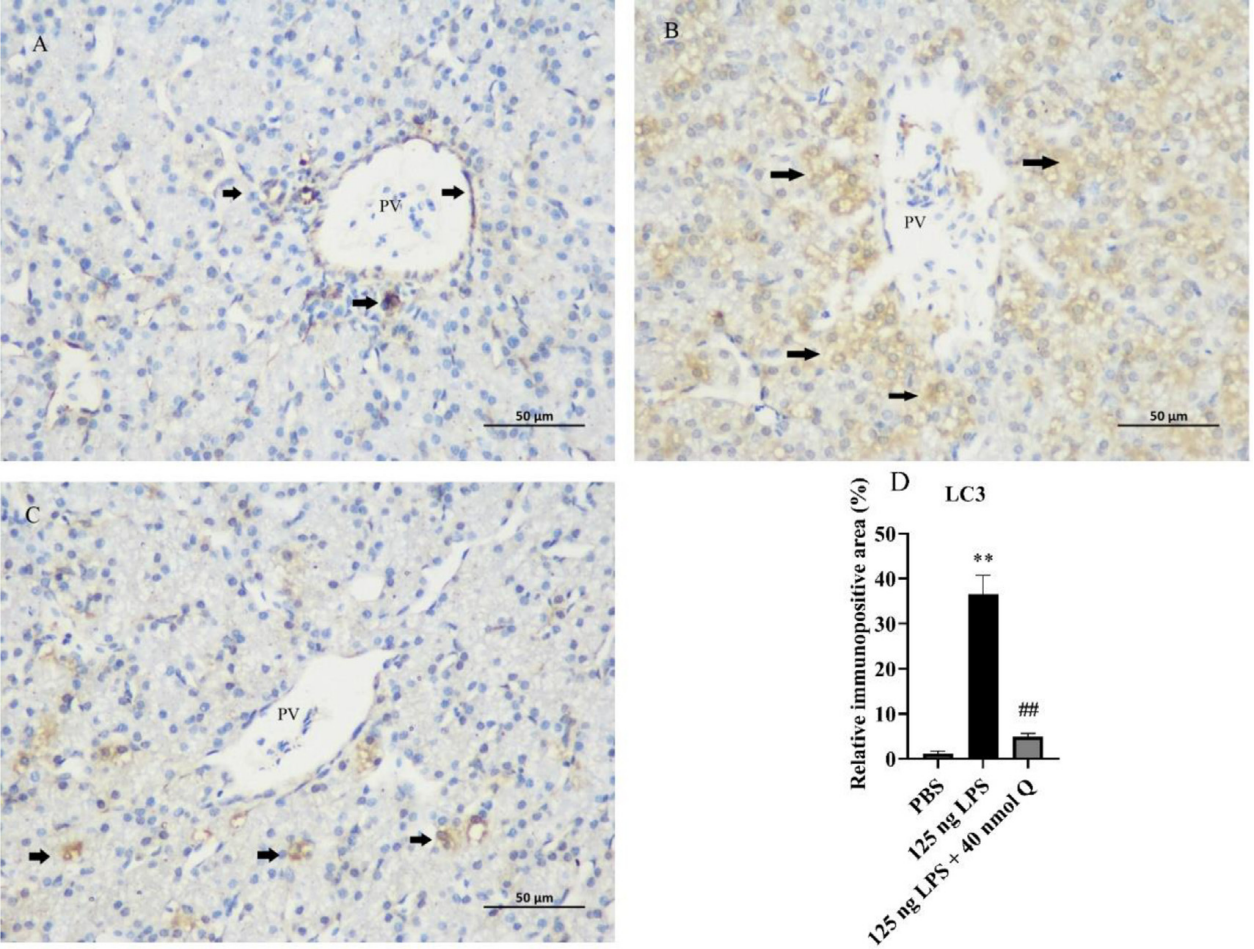
Quercetin alleviates the protein expression of LC3 induced by LPS in the livers of chicken embryos by immunohistochemical investigation. (A) PBS group; (B) LPS group (125 ng /egg); (C) treatment group (125 ng LPS/egg + 40 nmol quercetin /egg); (D) relative immunopositive area of LC3. Immunopositivity to LC3 (arrow, brown to yellow). Scale bar: 50 μm. Data were expressed as the mean ± SD. * indicates the significant differences between the PBS group and the LPS group; **: *P* < 0.01. # indicates the significant differences between the LPS group and the LPS + Q group; ##: *P* < 0.01.

**Figure 7:**
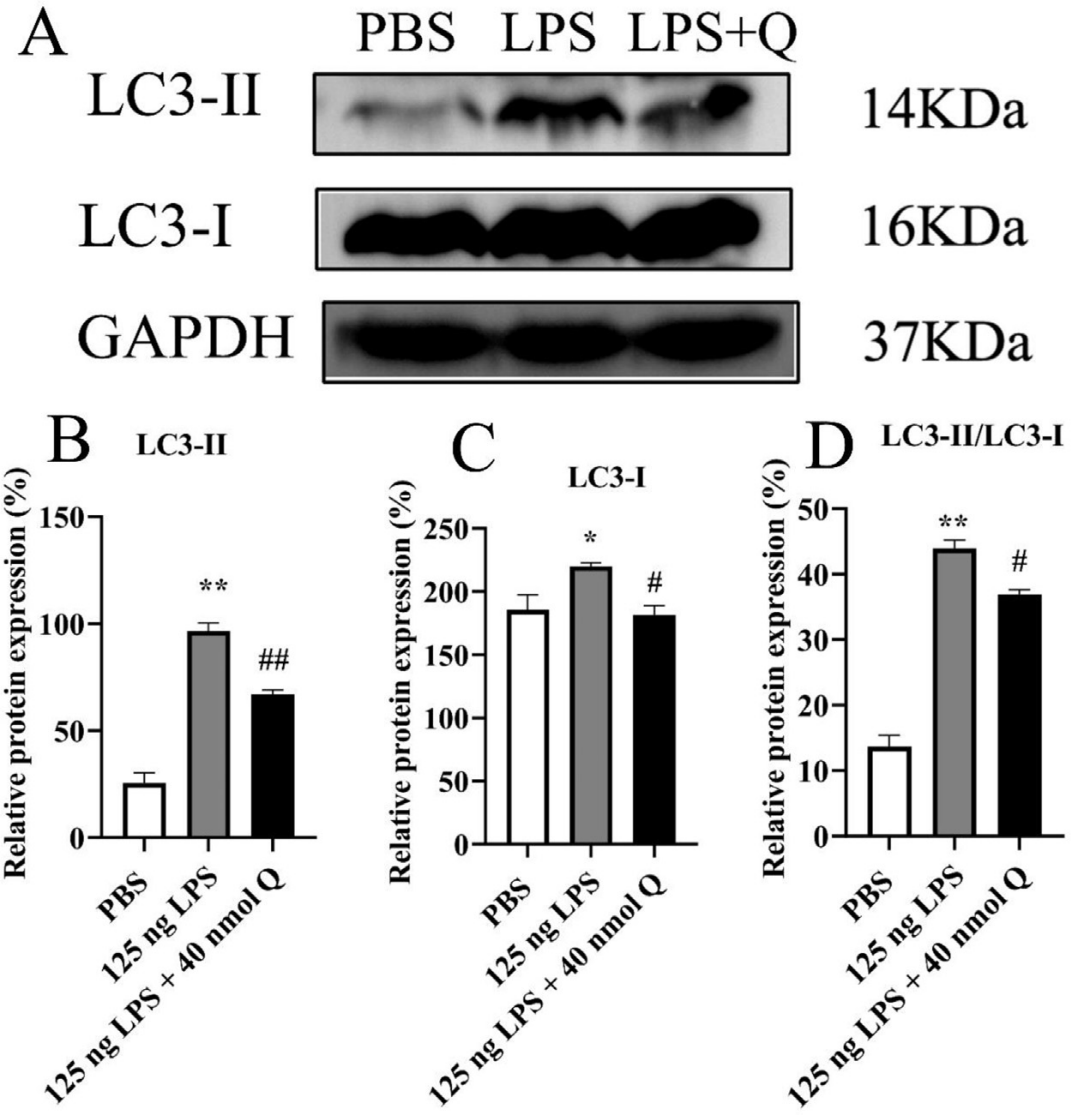
Quercetin alleviates protein expression levels of LC3-I and LC3-II after LPS induction in the livers of chicken embryos. PBS group; LPS group:125 ng LPS /egg; quercetin treatment group LPS + Q: (125 ng LPS + 40 nmol Q)/egg. A, the protein expression of LC3-I and LC3-II; B, relative protein expression of LC3-II; C, relative protein expression of LC3-I; D, ratio of relative protein expression of LC3-II/LC3-I. Data were expressed as the mean ± SD. * indicates the significant differences between the PBS group and the LPS group; # indicates the significant differences between the LPS group and the LPS + Q group.

The immunopositivity of LC3 (Figure 6) and protein expression of LC3-I, LC3-II, and rate of LC3-II/LC3-I (Figure 7) in the liver significantly increased after LPS induction when compared with that of the PBS group (*P* < 0.05 or *P* < 0.01), whereas quercetin treatment decreased the immunopositivity or protein expression of LC3 (*P* < 0.05 or *P* < 0.01).

## DISCUSSION

Acute liver injury is a clinical hallmark of endotoxemia regarding the features of septic organ failures. Hepatocytes are long-lived cells with little turnover. Toll-like receptors 4, one of pattern recognition receptors, are evolutionarily conserved family receptors that recognize the pathogen-associated molecular pattern (**PAMP**) such as LPS, bacteria, and virus which resulting in inflammatory cytokine storm. TLR4 plays a crucial role in the innate and adaptive immunity. It was found that the immunopositivity to TLR4 in the plasma of hepatocytes increased after LPS induction, and quercetin could decrease the protein expression of TLR4 when compared with LPS groups. LPS promote the TLR4-mediated MYD88 dependent signaling cascades, then stimulate the inflammatory factors (TNFα, IL-6, IL-8, and NF-κB1) expression. In this process, IL-6 and IL-10 are key contributors for eliciting pro-inflammatory responses (Kamimoto et al., 2009). One study found that the serum levels of IL-10 elevated in animals after portal venous *Escheriacia coli* infection (Jeyarajah et al., 2006). Plasma pro-inflammatory cytokines such as TNFα and IL-6 increased after LPS challenge in IL-10 knockout mice (Cordoba-Moreno et al., 2019). IL-6 is a major trigger for the synthesis of acute phase proteins by liver parenchymal cells. A previous study indicated that the mRNA expressions of IL-6 and NF-κB1 as key gene upregulated in the liver after LPS induction in Magang geese (Li et al., 2022). The present study indicated that the mRNA expression of TLR4, MYD88, NF-κB1, TNFα, IL-6, and IL-10 were upregulated after LPS induction in the livers of chicken embryos, similar to the results of a previous study (Khan et al., 2021). Meanwhile, quercetin could reduce the mRNA expression of TLR4, MYD88, NF-κB1, TNFα, IL-6, and IL-10. It suggested quercetin attenuated the LPS induced pro-inflammatory factors expression by inhibiting TLR4/MYD88/NF-κB1 signaling pathway in the livers of chicken embryos. TNFα and IL-6 can activate p38 signaling pathway which facilitates cell proliferation by translational regulation of protein synthesis. IL-1β is regulated through the activation of MAPK, including p38 as well as several transcription factors (Talwar et al., 2017).

LPS induced TNF binding to TNFRI and activated NF-κB1 and p38 mitogen-activated protein kinase pathways. The p38 pathway regulates cellular response to stress and inflammation, and is implicated in cell proliferation and differentiation. We found that the mRNA expressions of NF-κB1 and p38 in the PBS group decreased when compared with the control group. It might be associated with the hydrogen ion level of chicken embryos. Previous study revealed that the hydrogen ion level cumulated and increased with hatch age for chicken embryos (Murray and Assistance of Yetta, 1926), and another study indicated that the hydrogen-rich saline treatment significantly decreased the p38 MAPK mRNA expression and p-p38 MAPK protein level in the placentas of pregnant rats (Guo et al., 2023). Therefore, the hydrogen ion level increased when the PBS injected into the chick embryos, it significantly decreased the mRNA expression of p38. Phosphorylated p38 mitogen-activated protein kinase, which plays a major role in production of TNFα at the posttranscriptional level. One study found that both p38 alpha and IkappaB kinase 2 deletion in hepatocyte of mice presented liver failure after LPS induction (Heinrichsdorff et al., 2008). Another study showed that a murine sepsis model induced by cecal ligation and puncture stimulated NF-κB1, TNFα, and IL-6 activation in the livers (Williams et al., 1999). One study indicated that the mRNA expression level of IL-6 and p38 increased after LPS stimulation in rat liver (Tang et al., 2015). Hepatocytes, sinusoidal endothelial cells, and kupffer cells are the first cell populations in the liver that come into contact with gut-derived endotoxin in portal blood. One study indicated that the LPS stimulated the sinusoidal endothelial cells and kupffer cells excreted IL-6 with dose-dependent (Knolle et al., 1997).

MYD88, a central adaptor of innate immunity, mediates energy, synthesis of bioactive lipids and bile acids, and glucose metabolism (Duparc et al., 2017). Mice with MYD88 deletion were predisposed to glucose intolerance, liver fat accumulation, and inflammation (Lefort et al., 2019). Our present study indicated the mRNA expression of MYD88 downregulated with quercetin treatment, it might be associated with the bile acids synthesis and fatty acids metabolism. Because the saturated fatty acid decreased with chicken embryos age, while unsaturated fatty acid increased as the embryos grew elder in normal physical condition (Gomez-Munoz and Rodriguez-Fernandez, 1988); however, quercetin, an inhibitor of fatty acid synthase (Xia et al., 2023), could suppress the synthesis of the fatty acid, and reduced fatty acid affected and decreased the mRNA expression of MYD88 in TLR4/MYD88 signaling pathway.

The hepatic extracellular matrix is a complex network of macromolecules that not only provides cells with an extracellular scaffold but also plays a vital role in the regulation of cellular activities. The unbalance between the generation and degradation of extracellular matrix leads to collagen accumulation, and eventually develops into liver fibrosis. Evidence showed that the LPS-induced protein expression of MMP3 elevated in the microglia (Shi et al., 2014) and human microvascular endothelial cells (Kadry et al., 2021). However, there is no relative report on LPS-induced expression of MMP3 in the liver. The results of this study indicated that the hepatic mRNA and protein expression of MMP3 increased after LPS induction. It meant the degradation of extracellular matrix increased after LPS induction.

Autophagy is a pivotal and conserved cellular degradation process in eukaryotes. ATG5, ATG7, LC3-I, and LC3-II were biomarkers of hepatic conventional autophagy, which are vital components for the formation of autophagosome. Proliferation and regeneration capacity of ATG7-deficient hepatocytes was impaired (Romermann et al., 2020). Autophagy promoted hepatocellular proliferation (Pi et al., 2021). It was found that the autophagy-related mRNA expression of ATG5, ATG7, LC3A, and LC3B increased after LPS stimulation in the liver of chicken embryos, and quercetin could downregulate the mRNA expression of ATG5, ATG7, LC3A, and LC3B. In addition, quercetin could downregulate the mRNA expression of ATG5 and LC3B in the liver of chicken embryos when compared the PBS + ethanol group with the quercetin group. Recent study demonstrated that berberine inhibited autophagy in activated hepatic stellate cells and in mice with liver fibrosis (Tan et al., 2023), consistent with our result. Bcl-2 binds to the essential autophagy factor Beclin-1 to enhance autophagy. Beclin-1 is an important autophagy protein, and provides a vital scaffold to form class Ⅲ phosphatidylinositol 3 kinase (PI3KC3) complexes for initiation of autophagosome formation and maturation.

Our finding indicated that the hepatic mRNA expression of Beclin-1 increased after LPS induction when compared with PBS group, meanwhile, quercetin also significantly upregulated the hepatic mRNA expression of Beclin-1 within 3 LPS + Q groups when compared with LPS group or PBS + ethanol group, respectively. It revealed both the LPS group and LPS + quercetin groups induced autophagy. A previous study found that the hydroxysafflor yellow A induced autophagy by promoting the expression of Beclin-1 in liver cancer cells (Chen et al., 2020), similar to our results. The reason remains unknown. The endoplasmic reticulum stress of liver occurred after LPS induction (Fu et al., 2023), cause aggregation of unfolded or misfolded proteins, and need to activate autophagy to deal with this situation. The mRNA expression of Beclin-1 might be associated with noncanonical autophagy (IL-22/beclin-1 dependent signaling pathway), because the mRNA expression of beclin-1 had no difference in control group, PBS group, PBS + ethanol group, quercetin group (10 nmol, 20 nmol, and 40 nmol, respectively). It was found that the higher expression of Beclin-1 may be associated with the IL-22. Previous study indicated that the mRNA expression of IL-22 increased 46.9 fold after 4 d infection with *Salmonella* Enteritidis (Matulova et al., 2013), and another study revealed that serum IL-22 concentration significantly increased in LPS-induced liver injury and activated autophagy (Shao et al., 2020); in addition, quercetin with 50mg/kg or 100mg/kg could increase the IL-22 level in the colon of female mice (Liu et al., 2020). Another study indicated that IL-22 could upregulate the expressions of Beclin-1 (Hu et al., 2016). Therefore, we hypothesized the mRNA expressions of IL-22 upregulated after LPS + quercetin induction and activated autophagy (the mRNA expressions of Beclin-1 increase). The underlying mechanism needs further research.

There was ATG5/ATG7-independent and alternative autophagy pathway. The Rab protein is a small GTPase that belongs to the Ras-like GTPase superfamily and regulates the vesicle traffic process (Ao et al., 2014). Unlike conventional autophagy, autophagosomes appeared to be generated in a Rab9-dependent manner by the fusion of the phagophores with vesicles derived from the trans-Golgi and late endosomes (Arakawa et al., 2017). Golgi Apparatus is an organelle in eukaryotic cells that stores and modifies proteins for specific functions and prepares them for transport to other parts of the cell. Golgi-associated Rab GTPases function is an important mediator in intracellular vesicle trafficking (Lu et al., 2021). The Rab protein is present in the plasma membrane, and early endosome that mediate fusion of endocytic vesicles to form early endosome. Rab5 activate vacuolar protein sorting 34 (Vps34) / beclin-1 /Vps15 complex which involved in autophagosome formation. In the present study, it was found that quercetin with LPS significantly upregulated the mRNA expression of beclin-1 when compared with LPS groups, the underlying reason remain unclear. However, another hypothesis that quercetin might activate the Rab5 and/or Rab9 in Golgi complex, it needs to further study. One study indicated that the notoginseng triterpenes improved the protein expression of Vps34 and beclin-1 (Huang et al., 2021b), similar to our results.

Autophagic pathways are involved in lipid homeostasis in liver functions. Energy deficiencies are compensated by lipophagy, intracellular organelles with neutral lipids broken down lipid droplets (Filali-Mouncef et al., 2022), the LC3-II and ATG7 were involved in lipophagy. The lipid (yolk) comprised 8 to 10% chicken embryos before hatch, lipid metabolism and rapid embryonic development was intensive and notable from embryonic d 15 to 21, and 80% of the yolk was mobilized and absorbed during this time (Noble and Cocchi, 1990). Lipid droplets are intracellular organelles that store neutral lipids as energy reservoir. One study indicated that hepatic ATG5 knockout mice could not induce hepatic biogenesis of lipid droplets (Sun et al., 2018). Another study demonstrated liver-specific ATG7 knockout mice abolished liver mitochondrial DNA segregation (Tostes et al., 2022). We found that a large quantity of cytoplasmic lipid droplets or vacuoles were accumulated in the hepatocytes of normal chicken embryos. There were morphological changes on cytoplasmic lipid droplets or vacuoles after LPS challenge, lipid droplets became smaller, fused and had vague outline, it suggested the energy need and lipolysis increased after LPS induction. This result was consistent with that of one study, they found that lipid consumption increased in liver of LPS-induced mice than fasted mice (Chung et al., 2017). Our previous study found that the hepatic mRNA expression of AMPKα1 and AMPKα2 increased after LPS challenge due to energy deficiencies (Yu et al., 2023), and the LC3-II and ATG7 increased in lipophagy. However, quercetin can improve the lipid metabolism and relieve the energy need.

Necroptosis is a newly uncovered form of programmed cell death with characteristics of both necrosis and apoptosis. The RIPK1, RIPK3, MLKL, and Drp1 are necroptosis-related factors. Recent studies uncovered that the hepatic mRNA (Gu et al., 2023) and protein expressions of RIPK1, RIPK3, MLKL, and Drp1 increased after LPS challenge in piglets (Xu et al., 2021), similar to our results. One study unveiled that the LC3 proteins were connected with RIPK1 and RIPK3 by LC3 interacting region (LIR) domain in the hypoxic myocardium and cardiomyocytes (Huang et al., 2021a). We found that the hepatic mRNA expression of Fas, Bcl-2, RIPK1, and Drp1 upregulated after LPS induction; meanwhile, quercetin could downregulated these expressions.

## CONCLUSIONS

In the present study, LPS could induce hepatic inflammation by triggering inflammatory cells infiltration, and upregulated the mRNA expressions of inflammation, autophagy, and necroptosis-associated genes, increase the protein expressions of TLR4, MMP3, and LC3; while quercetin alleviated hepatic inflammation and attenuated autophagy induced by LPS, and downregulated the mRNA expression of necroptosis-related genes.

## References

[bib0001] Ao X., Zou L., Wu Y. (2014). Regulation of autophagy by the Rab GTPase network. Cell Death Differ..

[bib0002] Arakawa S., Honda S., Yamaguchi H., Shimizu S. (2017). Molecular mechanisms and physiological roles of Atg5/Atg7-independent alternative autophagy. Proceed. Japn. Acad..

[bib0003] Chen Z., Liu L., Liu Y., Wang S., Zhang S., Dong R., Xu M., Ma Y., Wang J., Zhang Q., Wei P. (2020). Hydroxysafflor yellow A induces autophagy in human liver cancer cells by regulating Beclin 1 and ERK expression. Exp. Therap. Med..

[bib0004] Chung K.W., Kim K.M., Choi Y.J., An H.J., Lee B., Kim D.H., Lee E.K., Im E., Lee J., Im D.S., Yu B.P., Chung H.Y. (2017). The critical role played by endotoxin-induced liver autophagy in the maintenance of lipid metabolism during sepsis. Autophagy.

[bib0005] Cordoba-Moreno M.O., Todero M.F., Fontanals A., Pineda G., Daniela M., Yokobori N., Ramos M.V., Barrientos G., Toblli J.E., Isturiz M.A., Rearte B. (2019). Consequences of the Lack of IL-10 in different endotoxin effects and its relationship with glucocorticoids. Shock.

[bib0006] Dou X., Yan D., Liu S., Gao L., Shan A. (2022). Thymol alleviates LPS-induced liver inflammation and apoptosis by inhibiting NLRP3 inflammasome activation and the AMPK-mTOR-autophagy pathway. Nutrients.

[bib0007] Duan G., Huang P., Zheng C., Zheng J., Yu J., Zhang P., Wan M., Li F., Guo Q., Yin Y., Duan Y. (2023). Development and recovery of liver injury in piglets by incremental injection of LPS. Antioxidants.

[bib0008] Duparc T., Plovier H., Marrachelli V.G., Van Hul M., Essaghir A., Stahlman M., Matamoros S., Geurts L., Pardo-Tendero M.M., Druart C., Delzenne N.M., Demoulin J.B., van der Merwe S.W., van Pelt J., Backhed F., Monleon D., Everard A., Cani P.D. (2017). Hepatocyte MyD88 affects bile acids, gut microbiota and metabolome contributing to regulate glucose and lipid metabolism. Gut.

[bib0009] Estonius M., Karlsson C., Fox E.A., Hoog J.O., Holmquist B., Vallee B.L., Davidson W.S., Jornvall H. (1990). Avian alcohol dehydrogenase: the chicken liver enzyme. Primary structure, cDNA-cloning, and relationships to other alcohol dehydrogenases. Eur. J. Biochem..

[bib0010] Fan X., Yang X., Guo N., Gao X., Zhao Y. (2023). Development of an endoplasmic reticulum stress-related signature with potential implications in prognosis and immunotherapy in head and neck squamous cell carcinoma. Diagn. Pathol..

[bib0011] Filali-Mouncef Y., Hunter C., Roccio F., Zagkou S., Dupont N., Primard C., Proikas-Cezanne T., Reggiori F. (2022). The menage a trois of autophagy, lipid droplets and liver disease. Autophagy.

[bib70] Fu J.N., Liu S.C., Chen Y., Zhao J., Lu N. (2023). Forsythiaside A Alleviates Lipopolysaccharide-Induced Acute Liver Injury through Inhibiting Endoplasmic Reticulum Stress and NLRP3 Inflammasome Activation. Biol. Pharm. Bull..

[bib0012] Gomez-Munoz A., Rodriguez-Fernandez C. (1988). Fatty acid composition of liver lipids during ontogeny of the chick embryo: effect of a single dose of triiodothyronine. Exp. Clin. Endocrinol..

[bib0013] Gu K., Wang F., Sun W., Liu G., Jia G., Zhao H., Chen X., Wu C., Tian G., Cai J., Zhang R., Wang J. (2023). Tryptophan alleviates lipopolysaccharide-induced liver injury and inflammation by modulating necroptosis and pyroptosis signaling pathways in piglets. Anim. Biotechnol..

[bib0014] Guo L., Liu M., Duan T. (2023). Hydrogen suppresses oxidative stress by inhibiting the p38 MAPK signaling pathway in preeclampsia. Adv. Clin. Exp. Med..

[bib0015] Hagiwara S., Iwasaka H., Hasegawa A., Kudo K., Kusaka J., Oyama Y., Noguchi T. (2012). Infusion of a glucose solution reduces autophagy in the liver after LPS-induced systemic inflammation. Inflammation.

[bib0016] Heinrichsdorff J., Luedde T., Perdiguero E., Nebreda A.R., Pasparakis M. (2008). p38 alpha MAPK inhibits JNK activation and collaborates with IkappaB kinase 2 to prevent endotoxin-induced liver failure. EMBO reports.

[bib0017] Hendriksen R.S., Vieira A.R., Karlsmose S., Lo Fo Wong D.M., Jensen A.B., Wegener H.C., Aarestrup F.M. (2011.). Global monitoring of Salmonella serovar distribution from the World Health Organization Global Foodborne Infections Network Country Data Bank: results of quality assured laboratories from 2001 to 2007. Foodborne Pathogens Dis..

[bib0018] Hu M., Yang S., Yang L., Cheng Y., Zhang H. (2016). Interleukin-22 alleviated palmitate-induced endoplasmic reticulum stress in INS-1 cells through activation of autophagy. PLoS One.

[bib0019] Huang Y., Feng Y., Cui L., Yang L., Zhang Q., Zhang J., Jiang X., Zhang X., Lv Y., Jia J.Z., Zhang D.X., Huang Y.S. (2021). Autophagy-related LC3 accumulation interacted directly with LIR containing RIPK1 and RIPK3, stimulating necroptosis in hypoxic cardiomyocytes. Front. Cell Develop. Biol..

[bib0020] Huang Z., Luo X., Zhang Y., Ying Y., Cai X., Lu W., Zhao J., Wang Y., Lin W., Tu Y., Xiang Z., Wu Q., Yang S., Zhu S., Li X. (2021). Notoginseng triterpenes inhibited autophagy in random flaps via the beclin-1/VPS34/LC3 signaling pathway to improve tissue survival. Front. Bioeng. Biotechnol..

[bib0021] Jeyarajah D.R., Kielar M.L., Saboorian H., Karimi P., Frantz N., Lu C.Y. (2006). Impact of bile duct obstruction on hepatic E. coli infection: role of IL-10. Am. J. Physiol. Gastrointest. Liver Physiol..

[bib0022] Kadry R.W., Adil M.S., Newsome A.S., Somanath P.R. (2021). Cisatracurium attenuates LPS-induced modulation of MMP3 and junctional protein expression in human microvascular endothelial cells. Biosci. Trends.

[bib0023] Kamimoto M., Mizuno S., Nakamura T. (2009). Reciprocal regulation of IL-6 and IL-10 balance by HGF via recruitment of heme oxygenase-1 in macrophages for attenuation of liver injury in a mouse model of endotoxemia. Int. J. Mol. Med..

[bib0024] Khan H.U., Aamir K., Jusuf P.R., Sethi G., Sisinthy S.P., Ghildyal R., Arya A. (2021). Lauric acid ameliorates lipopolysaccharide (LPS)-induced liver inflammation by mediating TLR4/MyD88 pathway in Sprague Dawley (SD) rats. Life Sci..

[bib0025] Knolle P.A., Loser E., Protzer U., Duchmann R., Schmitt E., zum Buschenfelde K.H., Rose-John S., Gerken G. (1997). Regulation of endotoxin-induced IL-6 production in liver sinusoidal endothelial cells and Kupffer cells by IL-10. Clin. Exp. Immunol..

[bib0026] Lefort C., Van Hul M., Delzenne N.M., Everard A., Cani P.D. (2019). Hepatic MyD88 regulates liver inflammation by altering synthesis of oxysterols. Am. J. Physiol.

[bib0027] Li B., Hong L., Luo Y., Zhang B., Yu Z., Li W., Cao N., Huang Y., Xu D., Li Y., Tian Y. (2022). LPS-induced liver injury of Magang Geese through toll-like receptor and MAPK signaling pathway. Animals.

[bib0028] Liu L., Liu Y., Cheng X., Qiao X. (2021). The alleviative effects of quercetin on cadmium-induced necroptosis via inhibition ROS/iNOS/NF-kappaB pathway in the chicken brain. Biol. Trace Element Res..

[bib0029] Liu W., Zhou Y., Qin Y., Yu L., Li R., Chen Y., Xu Y. (2020). Quercetin intervention alleviates offspring’s oxidative stress, inflammation, and tight junction damage in the colon induced by maternal fine particulate matter (PM(2.5)) exposure through the reduction of bacteroides. Nutrients.

[bib0030] Lu Q., Wang P.S., Yang L. (2021). Golgi-associated Rab GTPases implicated in autophagy. Cell Biosci..

[bib0031] Manders T.T.M., Matthijs M.G.R., Veraa S., van Eck J.H.H., Landman W.J.M. (2021). Success rates of inoculation of the various compartments of embryonated chicken eggs at different incubation days. Avian Pathol..

[bib0032] Matulova M., Varmuzova K., Sisak F., Havlickova H., Babak V., Stejskal K., Zdrahal Z., Rychlik I. (2013). Chicken innate immune response to oral infection with *Salmonella enterica* serovar Enteritidis. Vet. Res..

[bib0033] Maugeri A., Calderaro A., Patane G.T., Navarra M., Barreca D., Cirmi S., Felice M.R. (2023). Targets involved in the anti-cancer activity of quercetin in breast, colorectal and liver neoplasms. Int. J. Mol. Sci..

[bib0034] Meng J., Li Y., Sun F., Feng W., Ye H., Tian T., Lei M. (2024). Salidroside alleviates LPS-induced liver injury and inflammation through SIRT1- NF-kappaB pathway and NLRP3 inflammasome. Iran. J. Basic Med. Sci..

[bib0035] Murray H.A., Assistance of Yetta P. (1926). Physiological ontogeny: A. chicken embryos. Xi. The Ph, chloride, carbonic acid, and protein concentrations in the tissues as functions of age. J. Gen. Physiol..

[bib0036] Noble R.C., Cocchi M. (1990). Lipid metabolism and the neonatal chicken. Progr. Lipid Res..

[bib0037] Pi Q.Z., Wang X.W., Jian Z.L., Chen D., Zhang C., Wu Q.C. (2021). Melatonin alleviates cardiac dysfunction via increasing Sirt1-mediated beclin-1 deacetylation and autophagy during sepsis. Inflammation.

[bib0038] Romermann D., Ansari N., Schultz-Moreira A.R., Michael A., Marhenke S., Hardtke-Wolenski M., Longerich T., Manns M.P., Wedemeyer H., Vogel A., Buitrago-Molina L.E. (2020). Absence of Atg7 in the liver disturbed hepatic regeneration after liver injury. Liver Int..

[bib0039] Sang R., Ge B., Li H., Zhou H., Yan K., Wang W., Cui Q., Zhang X. (2023). Taraxasterol alleviates aflatoxin B(1)-induced liver damage in broiler chickens via regulation of oxidative stress, apoptosis and autophagy. Ecotoxicol. Environ. Saf..

[bib0040] Shaker N.S., Sahib H.B. (2023). Fraxin in combination with dexamethasone attenuates LPS-induced liver and heart injury and their anticytokine activity in mice. Adv. Virol..

[bib0041] Shao L., Xiong X., Zhang Y., Miao H., Ren Y., Tang X., Song J., Wang C. (2020). IL-22 ameliorates LPS-induced acute liver injury by autophagy activation through ATF4-ATG7 signaling. Cell Death Dis..

[bib0042] Shen Y., Xiao Y., Zhang S., Wu S., Gao L., Shi S. (2019). Fe3O4 nanoparticles attenuated salmonella infection in chicken liver through reactive oxygen and autophagy via PI3K/Akt/mTOR signaling. Front. Physiol..

[bib0043] Shi F., Duan S., Cui J., Yan X., Li H., Wang Y., Chen F., Zhang L., Liu J., Xie X. (2014). Induction of matrix metalloproteinase-3 (MMP-3) expression in the microglia by lipopolysaccharide (LPS) via upregulation of glycoprotein nonmetastatic melanoma B (GPNMB) expression. J. Mol. Neurosci..

[bib0044] Soldatenko A., Hoyt L.R., Xu L., Calabro S., Lewis S.M., Gallman A.E., Hudson K.E., Stowell S.R., Luckey C.J., Zimring J.C., Liu D., Santhanakrishnan M., Hendrickson J.E., Eisenbarth S.C. (2022). Innate and adaptive immunity to transfused allogeneic RBCs in mice requires MyD88. J. Immunol..

[bib0045] Sun Y., Ma Y., Sun F., Feng W., Ye H., Tian T., Lei M. (2023). Astragaloside IV attenuates lipopolysaccharide induced liver injury by modulating Nrf2-mediated oxidative stress and NLRP3-mediated inflammation. Heliyon.

[bib0046] Sun Y., Yao X., Zhang Q.J., Zhu M., Liu Z.P., Ci B., Xie Y., Carlson D., Rothermel B.A., Sun Y., Levine B., Hill J.A., Wolf S.E., Minei J.P., Zang Q.S. (2018). Beclin-1-dependent autophagy protects the heart during sepsis. Circulation.

[bib0047] Talwar H., Bauerfeld C., Bouhamdan M., Farshi P., Liu Y., Samavati L. (2017). MKP-1 negatively regulates LPS-mediated IL-1beta production through p38 activation and HIF-1alpha expression. Cell. Signal..

[bib0048] Tan Y., Li C., Zhou J., Deng F., Liu Y. (2023). Berberine attenuates liver fibrosis by autophagy inhibition triggering apoptosis via the miR-30a-5p/ATG5 axis. Exp. Cell Res..

[bib0049] Tang J., Li L., Li C.M., Wu J., Sun Y., Wang G.L. (2015). Upregulation of HO-1 with haemin alleviates LPS-stimulated pro-inflammatory responses through downregulation of p38 signalling pathways in rat liver. Scand. J. Immunol..

[bib0050] ten Busch M., Milakofsky L., Hare T., Nibbio B., Epple A. (1997). Impact of ethanol stress on components of the allantoic fluid of the chicken embryo. Comp. Biochem. Physiol. Part A, Physiol..

[bib0051] Tostes K., Dos Santos A.C., Alves L.O., Bechara L.R.G., Marascalchi R., Macabelli C.H., Grejo M.P., Festuccia W.T., Gottlieb R.A., Ferreira J.C.B., Chiaratti M.R. (2022). Autophagy deficiency abolishes liver mitochondrial DNA segregation. Autophagy.

[bib0052] Wang D., Xie W., He W., Zhu H., Zhang Y., Gao Q., Cong X., Cheng S., Liu Y. (2023). Selenium-enriched cardamine violifolia alleviates LPS-induced hepatic damage and inflammation by suppressing TLR4/NODs-necroptosis signal axes in piglets. Biol. Trace Element Res..

[bib0053] Williams D.L., Ha T., Li C., Kalbfleisch J.H., Ferguson D.A. (1999). Early activation of hepatic NFkappaB and NF-IL6 in polymicrobial sepsis correlates with bacteremia, cytokine expression, and mortality. Ann. Surg..

[bib0054] Woh P.Y., Yeung M.P.S., Goggins W.B., Lo N., Wong K.T., Chow V., Chau K.Y., Fung K., Chen Z., Ip M. (2021). Genomic epidemiology of multidrug-resistant nontyphoidal salmonella in young children hospitalized for gastroenteritis. Microbiol. Spectr..

[bib0055] Wu L., Chen Q., Dong B., Geng H., Wang Y., Han D., Zhu X., Liu H., Zhang Z., Yang Y., Xie S., Jin J. (2023). Resveratrol alleviates lipopolysaccharide-induced liver injury by inducing SIRT1/P62-mediated mitophagy in gibel carp (Carassius gibelio). Front. Immunol..

[bib0056] Xia B., Li Y., Liu Y., Sun W., Chen J., Li L., Pang J., Liu X., Chen S., Cheng H. (2023). rapid separation of asiatic acid, quercetin, and kaempferol from traditional chinese medicine centella asiatica (L.) urban using HSCCC-semi-prep-HPLC and the assessment of their potential as fatty acid synthase inhibitors. Int. J. Anal. Chem..

[bib0057] Xiang Y., Zhang H., Zhang Z.X., Qu X.Y., Zhu F.X. (2022). Dihydrosanguinarine based RNA-seq approach couple with network pharmacology attenuates LPS-induced inflammation through TNF/IL-17/PI3K/AKT pathways in mice liver. Int. Immunopharmacol..

[bib0058] Xiao K., Zhou M., Lv Q., He P., Qin X., Wang D., Zhao J., Liu Y. (2023). Protocatechuic acid and quercetin attenuate ETEC-caused IPEC-1 cell inflammation and injury associated with inhibition of necroptosis and pyroptosis signaling pathways. J. Anim. Sci. Biotechnol..

[bib0059] Xu Q., Guo J., Li X., Wang Y., Wang D., Xiao K., Zhu H., Wang X., Hu C.A., Zhang G., Liu Y. (2021). Necroptosis underlies hepatic damage in a piglet model of lipopolysaccharide-induced sepsis. Front. Immunol..

[bib0060] Yang X., Huang J., Zhang Y., Liu S., Chen L., Xiao C., Zeng H., Wei X., Gu Q., Li Y., Wang J., Ding Y., Zhang J., Wu Q. (2020). Prevalence, abundance, serovars and antimicrobial resistance of Salmonella isolated from retail raw poultry meat in China. Sci. Total Environ..

[bib0061] Yu C.Y., Chou S.J., Yeh C.M., Chao M.R., Huang K.C., Chang Y.F., Chiou C.S., Weill F.X., Chiu C.H., Chu C.H., Chu C. (2008). Prevalence and characterization of multidrug-resistant (type ACSSuT) Salmonella enterica serovar Typhimurium strains in isolates from four gosling farms and a hatchery farm. J. Clin. Microbiol..

[bib0062] Yu J., Hu G., Cao H. (2022). Quercetin ameliorates lipopolysaccharide-induced duodenal inflammation through modulating autophagy, programmed cell death and intestinal mucosal barrier function in chicken embryos. Animals.

[bib0063] Yu J., Hu G., Guo X., Cao H. (2023). Quercetin alleviates inflammation and energy deficiency induced by lipopolysaccharide in chicken embryos. Animals.

[bib0064] Zeng Y.B., Xiong L.G., Tan M.F., Li H.Q., Yan H., Zhang L., Yin D.F., Kang Z.F., Wei Q.P., Luo L.G. (2019). Prevalence and antimicrobial resistance of *Salmonella* in pork, chicken, and duck from retail markets of China. Foodborne Pathog. Dis..

[bib0065] Zhang H., Xiang Y., Huang Y., Liang B., Xu X., Xie J., Du X., Yang C., Liu H., Liu H., Wang H., Yang M., Wang L., Hu X., Jin L., Li J., Jiang Y., Qiu S., Song H. (2021). Genetic characterization of mcr-1-positive multidrug-resistant *Salmonella enterica Serotype Typhimurium* isolated from intestinal infection in children and pork offal in China. Front. Microbiol..

[bib0066] Zhao H., Liu H., Yang Y., Wang H. (2022). The role of autophagy and pyroptosis in liver disorders. Int. J. Mol. Sci..

[bib0067] Zhirong Z., Qiaojian Z., Chunjing X., Shengchen W., Jiahe L., Zhaoyi L., Shu L. (2021). Methionine selenium antagonizes LPS-induced necroptosis in the chicken liver via the miR-155/TRAF3/MAPK axis. J. Cell. Physiol..

[bib0068] Zhou X., Li X., Yi K., Liang C., Geng S., Zhu J., Xie C., Zhong C. (2022). Magnesium isoglycyrrhizinate ameliorates lipopolysaccharide-induced liver injury by upregulating autophagy and inhibiting inflammation via IL-22 expression. Bioorg. Chem..

[bib0069] Zhou Y., Hu X., Zhong S., Yu W., Wang J., Zhu W., Yang T., Zhao G., Jiang Y., Li Y. (2022). Effects of continuous LPS induction on oxidative stress and liver injury in weaned piglets. Vet. Sci..

